# The problem of energy poverty in the activities of agricultural advisory centres in Poland

**DOI:** 10.1371/journal.pone.0258366

**Published:** 2021-10-08

**Authors:** Arkadiusz Piwowar

**Affiliations:** Faculty of Economics and Finance, Wroclaw University of Economics and Business, Wrocław, Poland; Szechenyi Istvan University: Szechenyi Istvan Egyetem, HUNGARY

## Abstract

It is necessary in agricultural consulting to take into account the current problems as well as economic and social challenges facing rural areas. Undoubtedly, sustainable economy and energy policy is such a problem in Poland, including the issue of access to energy from renewable sources and financial possibilities of meeting the electricity demand of households and agriculture. Therefore, advisory and information activities in the field of reducing energy poverty and improving air quality are important. The main purpose of the paper was to identify and assess the role of advisory entities in the process of counteracting energy poverty in rural areas in Poland. The basic research method was an expert (survey) study. Research shows that the subject of energy poverty is relatively rarely taken up by consulting institutions as part of training courses, especially issues related to saving electricity in the household/agricultural sector are marginalized; monitoring and analysis of energy consumption; selection of technical devices in terms of energy efficiency. Based on expert research, there were identified consultancy activities that are and may be important in the topic under study. Experts indicated co-financing of activities in the field of energy efficiency as the preferred way to fight energy poverty in agriculture and rural areas. The results may constitute an important direction in the development of consultancy, the basis for building priority programs, which in turn may affect the behaviour and actions of farmers and other inhabitants of rural areas in the context of energy transformation. The performed research may constitute the basis for further, in-depth analyses in other countries and on an international scale.

## 1. Introduction

Rural areas are the areas particularly exposed to the problem of energy poverty. This is due to the relatively lower incomes of households located in rural areas compared to those in urban areas as well as the specific energy needs of the farmer’s household (for agricultural use) [1,2]. High energy sensitivity and vulnerability to energy poverty in rural areas is largely due to the socio-economic characteristics of rural households, the level of energy infrastructure development in this area and the characteristics of the apartment/house (building area and age, heat sources used, energy efficiency farm equipment). On the other hand, in rural areas, it is possible to use more progress in the field of biomass, hydropower and other renewable sources, which may help to improve the security of households in terms of the security of electricity supply [3]. The agricultural sector in Poland can plays a strategic role in ensuring Poland’s energy security [4]. The production and use of renewable energy sources are an important factor in minimizing the amount of harmful gases and dust emitted into the atmosphere, which are a consequence of the traditional combustion of fossil fuels [5]. It is possible to guarantee a high quality of life in rural areas (in terms of access and use of electricity) with rational use of available renewable resources [6].

The results of own research presented in this paper refer to information support and advice within the scope of energy poverty. Middlemiss et al. [7] indicate that social relations are a potential area supporting the solution of the problem of energy poverty. Relationships with family and friends are mainly indicated. According to the author, agricultural advisory services also have great potential in this regard and may play an important role in initiating or supporting actions taken to reduce the problem of energy poverty. Additionally, activities undertaken by institutions such as agricultural advisory centres should motivate farmers to introduce innovative technologies improving energy efficiency. The purpose of the activities of these organizations may be to coordinate issues related to the creation of groups of farmers and virtual networks in the topic of disseminating energy-saving technology. The basic issue is also raising awareness and educating the rural society on the issues of rational use of energy resources. The influence of agricultural advisors on the behaviour of farmers in terms of adopting new practices in Poland is large. This is indicated by previous studies, including in the area of innovation on the markets of agricultural inputs [8,9]. The main purpose of the paper was to identify and assess the role of advisory entities in the process of counteracting energy poverty in rural areas in Poland.

The article is structured as follows: after the introductory and methodological parts, section 2 presents the results of empirical research and discussion. In the fourth chapter, on the basis of statistical data, an outline of the problem of economic and energy poverty in rural areas in Poland is presented. The article ends with conclusions and recommendations for further actions and research.

## 2. Literature review

In the literature on the subject, the concept of energy poverty has many definitions, and its measurement may take place through the use of many indicators (LIHC, TPR, HEO, DCEN index, etc.) [10–15]. In general, energy poverty refers to situations where there are problems with maintaining an appropriate (comfortable) temperature in the place of residence (heating in winter and cooling in summer) and other problems related to lighting, cooking, using appliances [16]. The inability to achieve the basic standard of the level of energy services in the place of residence may result from the financial situation (price of energy/gas and payment capacity of households) and technical conditions (energy efficiency of the building and equipment). The phenomenon of energy poverty is characterized by the interdependence and interpenetration of socio-demographic as well as technical and economic factors with macroeconomic ones (e.g. labour market inefficiencies) [17–20]. According to Che et al. [21], energy availability and affordability are the major obstacles to alleviating energy poverty.

Energy poverty is a serious socio-economic challenge not only in countries with a relatively low level of socio-economic development, but also in highly and very highly developed countries (according to the human development index). Providing citizens with safe, sustainable access to energy at affordable prices is a policy priority of many countries and institutions. This issue is gaining importance in the public discourse and in political programs [17,22,23]. Energy poverty directly affects the quality of life and health, and has a significant impact on the environment [24–28]. Increasingly, attention is paid to the links between energy poverty and the harmful effects on mental health [29–31].

As Karpińska and Śmiech [32] emphasize, Poland is an interesting case study in the field of energy poverty. This is due both to insufficient research into the causes and extent of poverty, from a scientific point of view, and to worrying air pollution indicators. Poland is a country where electricity is mainly generated by burning hard coal and lignite. This method of generating energy has a significant negative impact on the environment due to the emissions generated during the combustion of coal. A significant problem for the air condition is pollution with suspended dusts (PM10 and PM2.5). In recent years, air quality standards have been systematically exceeded almost everywhere in Poland [33]. In the literature on the subject, apart from two types of smog (from London and Los Angeles), the term “*Polish smog*” also appears [34]. Also in other Central and Eastern European countries, such as Macedonia, Lithuania and Bulgaria, the number of households experiencing energy poverty is high [35].

The chance to improve the situation is perceived in the development of renewable energy sources. Investments in renewable energy sources can reduce energy costs and thus reduce the scale of energy poverty in Poland [36]. Renewable energy has a positive and statistically significant impact on the level of economic growth and development (including sustainable development) [37–39]. Relying on energy from renewable sources is also a huge benefit for the natural environment (reduction of greenhouse gases and pollutant emissions). The development of distributed and prosumer energy in Poland, however, faces problems related to installation costs [40].

The energy transformation in Poland, especially in rural areas, must be linked to socio-cultural and environmental changes at the local level. The research by Chodkowska-Miszczuk et al. [41] shows that it is necessary in rural areas to manage electricity and heat more ecologically. In conclusion, the authors emphasize that institutional support and strengthening of local social capital are important. It is crucial, especially in a situation where various forms of poverty coexist in rural areas. This underlines the importance of conducting research and analyses in the area of social and institutional determinants of the energy transformation in Poland.

## 3. Methodology and data

**Fig 1:**
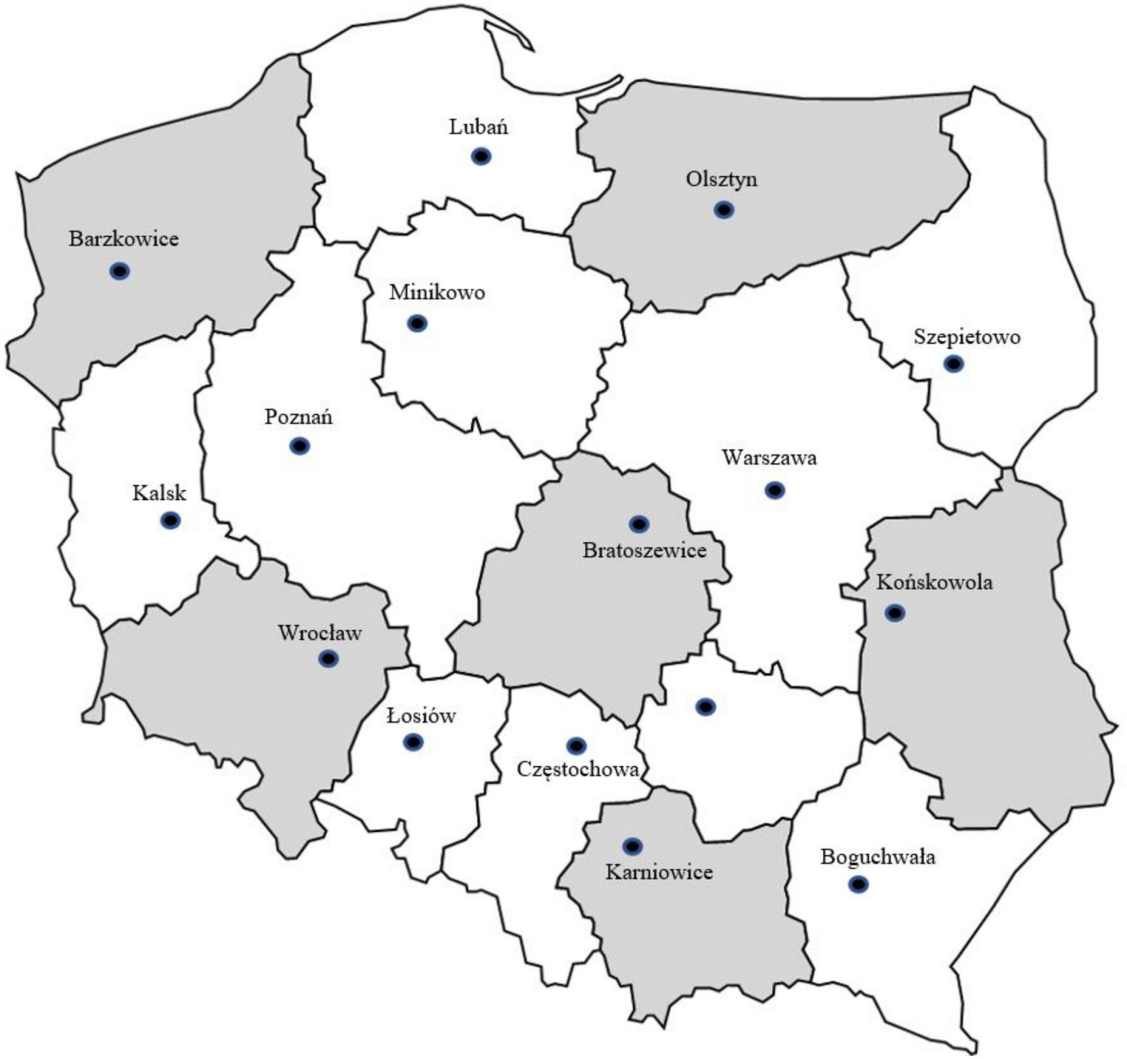
Seats of voivodeship agricultural advisory centres in Poland and location of the researched centres [42]. Source: Own elaboration base on [42].

The article uses both secondary and primary data. The methods of collecting data used in the research include the analysis of the literature on the subject and the survey method. The article was based on the author’s own research, carried out in the course of the research project financed by the National Science Centre in Poland. The above-described survey was one of the three main stages of research under this project. The remaining two stages (quantitative and qualitative research) concerned farmers’ households in the area of six randomly selected provinces (one from each macroregion in Poland). The sampling frame included all 16 provinces in Poland, and the following provinces were drawn: Dolnośląskie; Zachodniopomorskie; Lubelskie; Warmińsko-mazurskie; Małopolskie and Łódzkie (Fig 1). The spatial scope of the research of farmers and experts was identical. Adoption of the research methodology taking into account the division into macro-regions and random selection in their spatial scope made it possible to consider various socio-economic features of rural areas in Poland.

The analyses used the results of the survey conducted between November 2019 and March 2020. The selection of the sample for expert research was deliberate. The research project established that the minimum research sample would be 30 experts, including at least 4 experts with knowledge and experience in the field of energy poverty in the study area. The experts were mainly employees of agricultural advisory centres. The research was carried out on the basis of an appropriate agreement between the University (Wroclaw University of Economics and Business) and the Directorate of Agricultural Advisory Centres in the selected provinces. The anonymous questionnaires, constituting an attachment to the above-mentioned agreement, were sent back by traditional mail to the research project manager (the author of this work). All study participants provided oral informed consent, and the study design was approved by the appropriate ethics review board. The Rector’s Committee for Ethics of Scientific Research at the University of Economics and Business in Wrocław, based on the application of the author of the work (research project manager), issued a positive opinion on ethical questions (Application no. 57/2020). The IRB specifically approved the study. The participants of the study gave oral consent to the survey. The ethics committee approved the consent procedures and the use of oral consent.

State institutions (agricultural advisory units) have the greatest traditions and experience in advisory activities in rural areas in Poland. These are units that constitute the main element of the Polish Farming Advisory System (FAS), with over 100 years of tradition in Poland. It was in these units that the research was carried out. In each of the 16 voivodeships in Poland, there are such institutions supporting the development of rural areas and agriculture, and a team of advisers operates in each district. The experts’ surveys will be carried out using the proprietary questionnaire. The research was conducted among 31 experts. The average age of the respondents was 49 years, and the experts were very experienced in the field of agriculture and rural development. 19 respondents declared experience in this field for over 20 years, and 9 respondents between 11–20 years. 30 respondents had higher education, including 27 in the field of agriculture.

## 4. Economic and energy poverty in rural areas in Poland—The outline and scale of the problem against the EU

The phenomenon of energy poverty, and therefore the inability to provide the socially and materially required level of energy services in the household, occurs throughout Europe and also affects rural areas [43].

**Fig 2:**
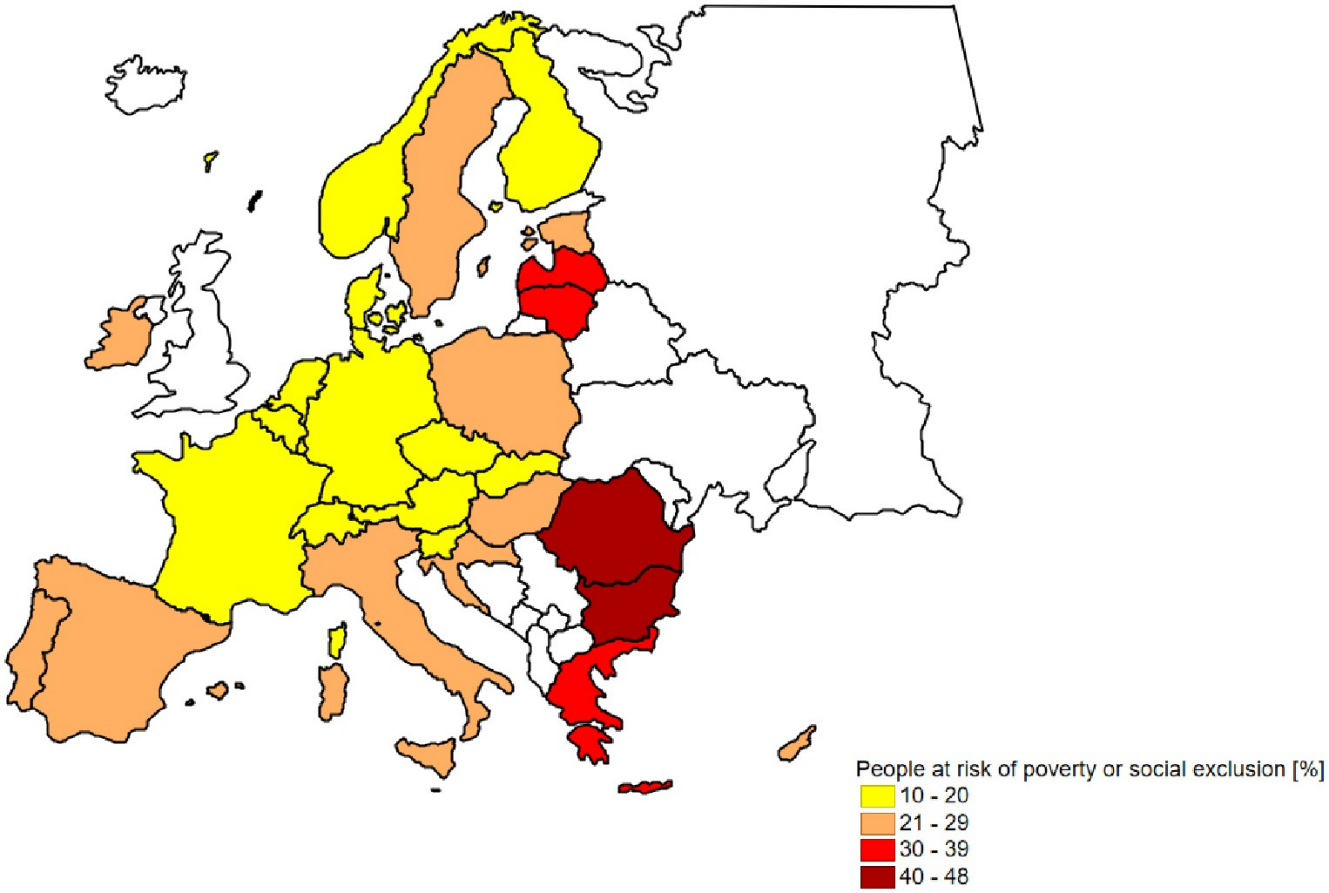
Share of people living in rural areas in UE-27 who are at risk of poverty or social exclusion in 2019 [44]. Source: Own elaboration base on [44].

The underlying phenomenon is economic poverty. Eurostat data show significant spatial differentiation in this respect. Poland belongs to the group of countries with a relatively average share of people affected by this problem living in rural areas. Nevertheless, in 2019, according to Eurostat data, 1,677 thousand people in Poland (24.1% of people living in rural areas) were affected by this problem (Fig 2).

The worst situation in this respect in the EU was in Romania and Bulgaria, where the rate exceeded 40%. Still a big problem in these countries is the very poor performance in terms of energy efficiency [45,46]. Taking into account the EU countries bordering Poland, both in Germany, the Czech Republic and Slovakia, indicators related to economic poverty in rural areas are lower. For instance, in the Czech Republic, less than 12% of people living in rural areas were affected by poverty and social exclusion. Moreover, the situation in the Czech Republic is so good that there is currently no official policy on energy poverty there [47]. This is largely due to the assistance programs operating in the Czech Republic in the area of the social benefits system, including e.g. housing costs [48].

Poverty, including energy poverty, is a highly spatially diversified phenomenon. The analysis should be based on the inclusion of local and regional factors in the assessment of energy poverty [49]. In Poland, 42% of citizens live in rural areas, which is well above the EU average. This fact also makes it worth analysing the group living in the Polish countryside. Statistical data obtained on the basis of the results of the survey of household budgets in Poland show that the level of extreme poverty in rural areas is higher than in cities (Table 1).

**Table 1:** The extent of extreme poverty in Poland in 2017–2019 (in% of people in households).

Specification	2017	2018	2019
By the class of the place of residence			
city (total)	2.4	2.8	2.1
> 500 thous.	1.5	0.9	1.0
200–500 thous.	1.1	1.2	1.4
100–199 thous.	1.8	2.7	2.1
20–99 thous,	2.8	3.1	2.5
< 20 thous.	4.1	5.1	2.8
rural area	7.3	9.4	7.5
By socio-economic groups			
self-employed	2.6	3.4	2.4
retirees	4.4	4.6	3.5
employees	3.3	4.7	3.6
pensioners	7.3	8.4	6.3
farmers	9.7	11	9.8

Source: Own elaboration base on [50].

Extreme poverty rate in Poland is much higher in rural areas than in cities. Likewise, extreme poverty among farmers is the highest of all socio-economic groups. Attention is drawn to the high percentage of energy poor farmers (and rural residents) in 2018. This is due to the coexistence of many factors, including deterioration of the economic situation in Polish agriculture in 2018 and an increase in the prices of energy carriers. The issue of relatively high poverty in the countryside in Poland should be perceived in the complex causes and structural, institutional, economic and individual conditions [51].

The above-presented data on poverty in the Polish countryside indicate the need for increased activities in the field of social policy. As emphasized by Sokołowski et al. [12], differences in energy poverty between urban and rural areas are attributed to housing conditions (size and standard of buildings) and sources of income. The problem of low standard of residential and farm buildings in Poland is described in the literature on the subject [52]. Additionally, there is a problem of burning low-quality fuels in the old generation of individual heating systems [53]. These furnaces not only use low-quality coal and wood, but also waste. An additional, very big problem associated with it is air pollution as a result of burning coal and waste (especially high concentrations of PM10 and PM2.5 in winter) [54]. Thus, the ideal solution for reducing poverty and social exclusion would be to combine energy modernization measures with appropriate social, regional and agricultural policies as well as sustainable national strategies and policies for energy transformation [55–57]. The issues related to energy poverty in rural areas in Poland can therefore be considered in a broader context, namely the importance of rural areas in the fight against climate change.

## 5. Results

Experts participating in the study answered a total of 15 substantive questions, the scope of which concerned the essence, causes and consequences of energy poverty in rural areas in Poland.

Respondents declared, among others, whether they had encountered the problem of energy poverty in their professional work. 17 respondents (i.e. 54.8% of the research sample) declared that they had encountered this problem directly in conversations with residents, and 4 respondents declared indirect contact as part of other implemented activities (programs). 10 respondents (i.e. 32.2% of the research sample) stated that there was not such a situation in the course of performing their official duties.

**Fig 3:**
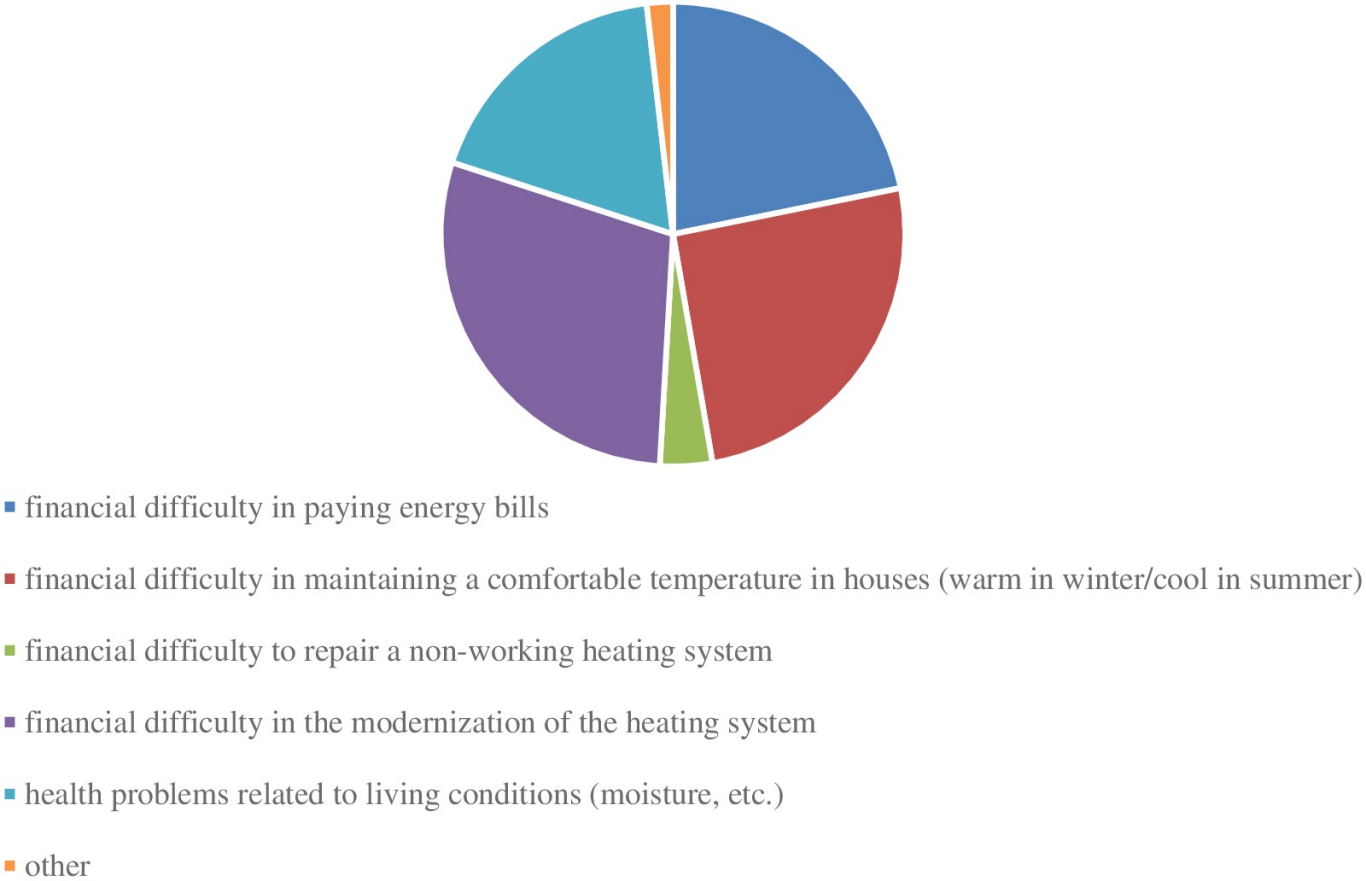
Main problems reported to agricultural advisors in the field of energy poverty in rural areas. Source: Own study based on questionnaire surveys (N = 16).

People who declared direct contact with a particular issue were asked to indicate the number of reported problems. Out of 17 respondents who declared this fact, 6 answered that these were “individual cases”; in turn, 8 respondents declared “a dozen or so cases”. According to the research, 3 experts marked “dozens of cases”. None of the respondents replied “hundreds or more cases”. In reference to the previous answers, the question was also asked about the reported problems in the examined area (multiple choice question). The results of this part of the research are presented in Fig 3.

**Fig 4:**
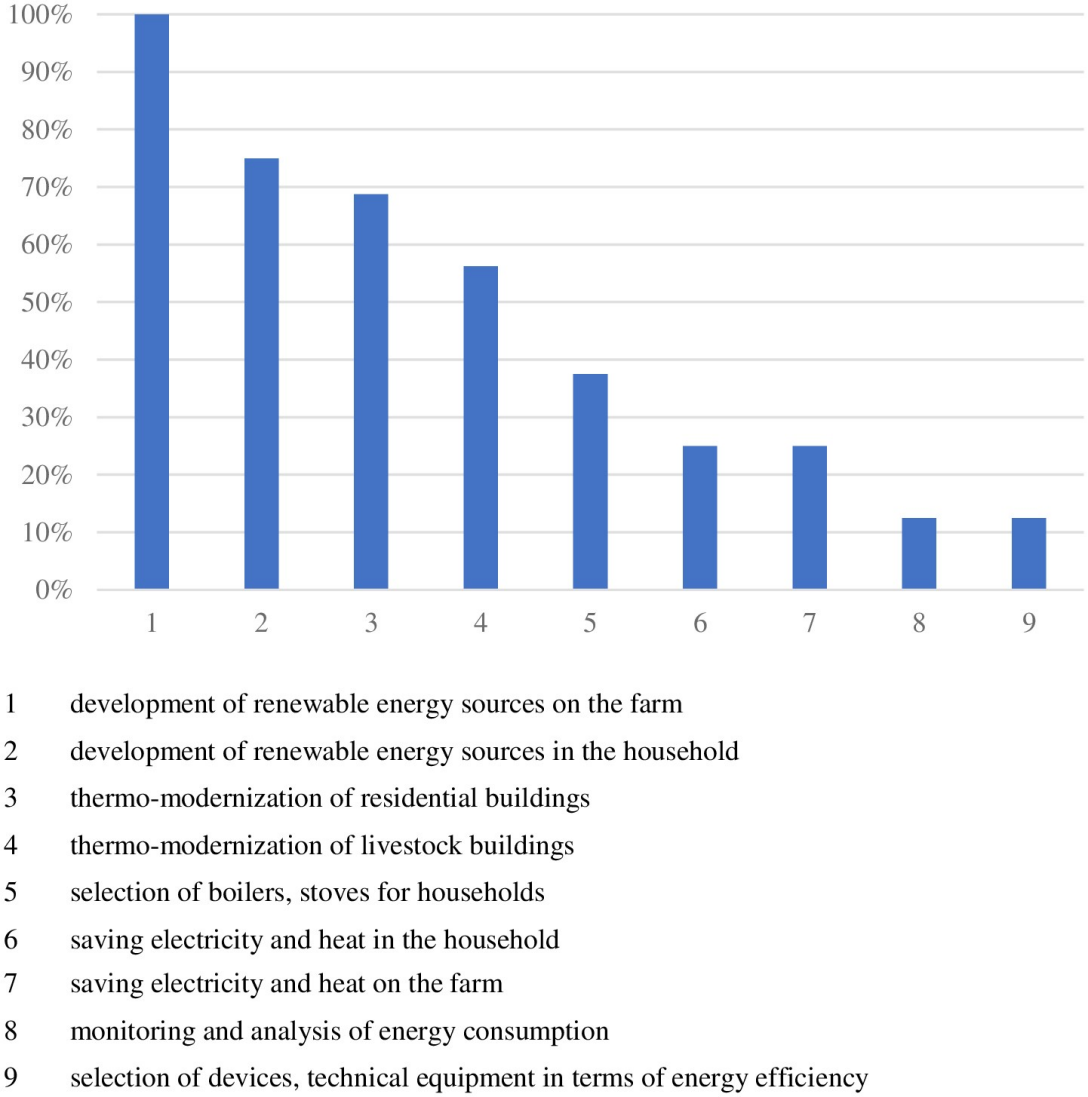
The most frequent topics of training in effective management of electricity and heat in the household of farmers/farm. Source: Own study based on questionnaire surveys (N = 16).

According to the research, 16 experts declared that effective management of electricity and heat in the household of farmers/farms was the subject of training in the institutions where they worked. Nevertheless, the research also obtained 15 declarations that such training was not carried out. Experts who declared the implementation of trainings in the particular scope indicated their subject (Fig 4).

All institutions, which implemented training in the studied topic, carried out the training on the development of renewable energy sources in farms (16 declarations). Popular topics of training were also: the development of renewable energy in households (12 indications) and thermal modernization of residential buildings (11 indications) and livestock buildings (9 indications). Other training topics, indicated in Fig 2, were marked by less than half of the respondents declaring training in the studied subject area.

The questions in the survey also concerned other issues related to the topic under study. When asked whether one of the priorities in RDP 2021–2027 should be the problem of energy efficiency of farms, 27 experts answered in the affirmative. A high percentage of affirmative responses was also obtained with regard to the questions whether a guide of good energy management practices in a farmer’s household and in a farm should be created (28 responses each).

Experts also had the opportunity to identify the most effective ways to combat energy poverty. For this purpose, the experts could rank the five factors listed in Table 2 on a scale of 1 (most important) to 5 (least important) according to their importance.

**Table 2:** Methods of combating energy poverty in rural areas in Poland preferred by the respondents.

Specification	Total points	Number of responses	Weighted Mean
Social tariffs (lower tariffs for low-income households)	84	30	2.8
Protection of households against disconnection from the power grid in winter	95	29	3.28
Co-financing of activities in the field of energy efficiency improvement (replacement of heat sources, thermal modernization, etc.)	61	31	1.97
Consulting (developing advisory programs for vulnerable groups of consumers, e.g. regarding the efficiency of energy use by households)	101	30	3.37
Lowering the costs of energy and heat supply by supporting the development of energy cooperatives/energy clusters based on local energy resources	92	30	3.07

Source: Own study based on questionnaire surveys (N = 31).

Experts indicated co-financing of activities in the field of energy efficiency (replacement of heat sources, thermo-modernization, etc.) as the preferred way to fight energy poverty (13 experts indicated as the most important way, the weighted average was 1.97). The remaining options were assessed relatively similarly by the respondents (weighted average from 2.8 to 3.37 points). Counselling was the lowest rated of the methods of combating energy poverty. Furthermore, less than half of the experts (15 people) admitted that they knew an example that could be described as good practice in the field of energy management in a farmer’s/farm household. The respondents were asked to indicate these good practices and most often (13 people) mentioned the use of solar energy for heating water (for consumption and production purposes). Two experts named agricultural biogas plants as examples of good practice in the rural areas where they operated.

In the course of the research, experts had the opportunity to propose tools to combat energy poverty that could be offered/supported at the central and local level. Experts mentioned various activities, including lowering taxes affecting the retail price of fuels and electricity, educational activities for inhabitants of rural areas, tax exemptions for farms modernizing systems towards greater use of renewable energy; facilitation of procedures for hydropower and support for energy cooperatives.

## 6. Discussion

Recently, regulations have been introduced in Poland to allow the deduction of expenditure on thermo-modernization of houses from income (the regulation from 2019). Thermo-modernization of houses in Poland is important as among 3.5 million houses heated with coal up to 2.2 million are low-energy buildings with low energy efficiency. This is also a significant problem in the context of the development of a low-carbon economy. The research problem undertaken in the work is additionally gaining importance due to the forecasted increase in electricity prices in Poland. Therefore, significant progress is required in expanding access to modern energy sources. In the literature on the subject, initiatives and policies aimed at solving this problem were proposed, among others, by Chakravarty and Tavoni [58]. Taking into account the example of Poland, it is necessary first of all to look at the problem of energy efficiency in single-family houses. The relatively low insulation performance of single-family buildings, with a relatively high percentage of coal heating, are the key elements necessary to change in order to reduce the emission of pollutants from the housing sector. It should be emphasized here that burning coal for heating purposes (and the relatively low individual costs associated with it) is related to high social costs. Therefore, social policy instruments should be developed and optimal conditions should be created for the development of general thermal modernization programs.

In Poland, the problems of energy poverty are mainly dealt with by independent research institutes, mainly the Institute for Structural Research (IBS) and the Institute for Sustainable Development. The reports of the Institute for Structural Research show that in 2019, 10% of the Polish population suffered from energy poverty. 380 thousand of energy poor households use coal or wood stoves [59]. Therefore, this is a real problem, more and more often discussed, but still not sufficiently recognized, especially in relation to households of farmers. To date, there is no detailed research at the microeconomic level in the literature on the subject, which would attempt to characterize the energy poverty of this group of households. However, the results of previous studies of the general level of energy poverty indicate that in Poland this problem mainly affects inhabitants of rural areas [12,60,61]. The available IBS reports show that every fifth person living in the countryside is struggling with energy problems. This is related to economic reasons (lower incomes of population in the countryside compared to city dwellers), and also to the fact that the villagers live in single-family houses, often of large square metrage and low energy efficiency. In addition, many people living in rural areas have no access to the heating and gas networks.

On the basis of these studies and the literature analysis of the subject, it is necessary to conclude about the relatively low use of energy-saving systems and devices both in the current functioning of the farm and the farmer’s household [62,63]. In some areas energy poverty is caused by the lack of resources, leading to the situation of multi-dimensional deprivation with different degrees of intensity, at different stages and phases. In households directly related to agriculture in Poland, i.e. the households of farmers and employees using farms, there is relatively small use of renewable energy sources [64–66]. The phenomenon of energy poverty in the countryside is also connected with other important issues—including health and air protection (also low emission issues) [67,68].

## 7. Conclusions

Energy poverty in the Polish countryside, due to its range, should be a key initiative assigned to the agricultural advisory system. This is due to the fact that the area of operation of this system is inextricably linked with the transfer of knowledge and innovation, which should solve the most important social and economic problems in rural areas. Energy poverty is undoubtedly such a problem, which results from official statistics and the results presented in scientific studies in this field. Every fifth rural inhabitant is affected by energy poverty and this group accounts for as much as two thirds of all those affected by this problem in Poland.

The subject of trainings related to the phenomenon of energy poverty should be included in the priorities of the advisory institutions operating in rural areas and included among the most important areas of intervention in the social area. The advisory needs of farmers and other rural residents in this respect seem to be unmet. It is worth emphasizing that the problem of energy poverty is a sensitive topic that may be the basis for discrimination or limit participation in professional and social life.

The results of the research can be used to formulate corrective actions and to create recommendations on the subject of this study, implementation and realization of training programs related to the issue of energy poverty in rural areas. Taking into account the policy and organization of agricultural advisory services in Poland, the recommendations concern both methodological issues (preparation of materials for agricultural advisors, training of advisers) and practical aspects (advising farmers and other rural residents, using innovative training methods—learning by doing, active learning). The subject of training should concern the broad context of the problem of energy poverty, including energy efficiency, modern energy services, aid programs aimed at reducing the scale of this phenomenon, etc. Due to the problem of low emission, it is also worth advising on the selection of modernization and investment activities improving efficient use of energy, leading to the reduction of CO_2_ emission and air pollution.

The obtained results can be used for further study, in particular in terms of analysing the content of emerging topics and training content, which will allow for further refinement of research methods and analytical frameworks. The limitation in the research being the basis of this work is the geographical scope of the field research. It is possible to extend the research to other voivodships in Poland and on this basis to conduct in-depth regional analyses. On the other hand, research can be focused on those districts (communes) where the problem of energy poverty in rural areas is the greatest. Additionally, the subjective scope of the research can be extended to include private consulting companies, which will allow for a more detailed overview of this area of activity of advisory entities in rural areas in Poland.
